# Midlife Aging and Performance Study: Adopting Physical Capacity Tests for Biological Age Assessment

**DOI:** 10.1093/geroni/igaf122.963

**Published:** 2025-12-31

**Authors:** Roy Tzemah-Shahar, Merav Asher, Maayan Agmon

**Affiliations:** University of Haifa, Haifa, HaZafon, Israel; University of Haifa, Haifa, HaZafon, Israel; University of Haifa, Haifa, HaZafon, Israel

## Abstract

Aging is a risk factor for the development of chronic diseases and functional decline; however, it is heterogenous. Measuring aging in midlife is commonly done using physiological markers to estimate Biological Age (BA). This method limits the possibilities of developing personalized behavioral interventions to promote healthier aging. To address this gap, we have developed the Midlife Aging and Performance Study (MAPS), a cross-sectional study aiming to examine the association between a comprehensive measure of physical capacity, relying on behavioral markers, and BA, measured by physiological markers. A total of 112 individuals (age 44.5±1.4, 47% women) were included in MAPS; BA was estimated using the Klemera-Doubal method. Physical capacity (PC) was measured using tests covering five behavioral domains (muscle strength, endurance, balance, agility, and flexibility). Higher performance in strength, endurance, balance and flexibility domains was correlated with younger BA (Pearson’s r 0.33-0.49, p < 0.001); a composite score of physical capacity, including five domains, was significantly negatively associated with accelerated aging in a logistic regression (where biological age>chronological age). The composite physical capacity score had an odds ratio of 0.40 (95% CI 0.25-0.64) in predicting accelerated aging, each incremental increase in physical capacity corresponded with a 60% reduction in the risk of being in an accelerated aging state. The proposed physical capacity battery also includes a functional behavioral assessment for aging state during midlife. Measuring physical capacity may help in population wide risk-screening assessment, presenting both easily conveyed intervention goals and a means to evaluate temporal changes of health independent of laboratory tests.

